# Mixing Performance Analysis and Optimal Design of a Novel Passive Baffle Micromixer

**DOI:** 10.3390/mi15020182

**Published:** 2024-01-26

**Authors:** Yiwen Zheng, Yu Liu, Chaojun Tang, Bo Liu, Hongyuan Zou, Wei Li, Hongpeng Zhang

**Affiliations:** 1 Marine Engineering College, Dalian Maritime University, Dalian 116026, China; evezywemail@163.com (Y.Z.); liuyu0717@dlmu.edu.cn (Y.L.); 13657757377@163.com (C.T.); 13674137039@163.com (B.L.); 17823114014@163.com (H.Z.); dmuliwei@dlmu.edu.cn (W.L.); 2 State Key Laboratory of Fluid Power and Mechatronic Systems, Zhejiang University, Hangzhou 310027, China

**Keywords:** passive micromixer, baffle, molecular diffusion, chaotic convection, numerical simulation

## Abstract

Micromixers, as crucial components of microfluidic devices, find widespread applications in the field of biochemistry. Due to the laminar flow in microchannels, mixing is challenging, and it significantly impacts the efficiency of rapid reactions. In this study, numerical simulations of four baffle micromixer structures were carried out at different Reynolds numbers (Re = 0.1, Re = 1, Re = 10, and Re = 100) in order to investigate the flow characteristics and mixing mechanism under different structures and optimize the micromixer by varying the vertical displacement of the baffle, the rotation angle, the horizontal spacing, and the number of baffle, and by taking into account the mixing intensity and pressure drop. The results indicated that the optimal mixing efficiency was achieved when the baffle’s vertical displacement was 90 μm, the baffle angle was 60°, the horizontal spacing was 130 μm, and there were 20 sets of baffles. At Re = 0.1, the mixing efficiency reached 99.4%, and, as Re increased, the mixing efficiency showed a trend of, first, decreasing and then increasing. At Re = 100, the mixing efficiency was 97.2%. Through simulation analysis of the mixing process, the structure of the baffle-type micromixer was effectively improved, contributing to enhanced fluid mixing efficiency and reaction speed.

## 1. Introduction

Microfluidics has significant advantages such as a lower sample requirement, faster analysis speed, less reagent consumption, and higher throughput, and is widely used in the fields of biology and medicine. As an important part of the microfluidic control chip, the micromixer has a broad application prospect in the fields of integration and miniaturization of the analytical system, chemical synthesis, and energy production [1,2,3].

According to whether there is an external energy field action, the micromixer can be divided into two categories, active and passive [4], in which the passive micromixer does not rely on external fields, more in line with the requirements of the microfluidic system in terms of convenience and integration. The single-channel micromixer has the advantages of a simple structure, low processing cost, and small pressure drop, and is most widely used in passive micromixers [5]. Commonly used single-channel micromixers include T-type [6], Y-type [7], sawtooth-type [8], square-wave-type [9], curved-channel-type [10,11], spiral-channel-type [12] and twisted-channel-type [13] etc. Because the T-type micromixer has a large number of baffles, the fluid flow path is longer, the vortex intensity is stronger, and it has a better mixing efficiency. At the same time, the T-shaped structure is relatively simple and easy to manufacture and integrate [14].

It is a common design to add baffles to micromixers [15,16,17,18]. The presence of baffles can guide the fluid to mix more effectively, helping to increase the mixing effect. By designing appropriate baffles, precise control of the degree of mixing can be achieved to meet the requirements of specific applications, reduce inhomogeneities in fluid flow, and ensure more uniform mixing [19,20,21,22]. Ansari et al. [23] conducted experiments and numerical analyses on the design of a micromixer that generates vortex flow, and compared the mixing performance with a simple micro T-type mixer, and further extended it to planar serpentine microchannels. These microchannels combined with simple and swirling T-junctions to evaluate and validate their mixing performance. The mixing performance of the vortex T-type mixer is higher than that of the simple T-type mixer, and increases significantly with the increase of the Reynolds number. Ahmadi et al. [24] added baffles to the side walls of the curved snake-type micromixer. When the fluid flows through the baffles, chaotic convection is generated, which enhances fluid mixing. They also compared and optimized six different baffle structures through numerical research. Agarwal et al. [25] added a diffuser plate to a T-shaped micromixer and studied the effects of different arrangements of diffuser plates on mixing. The effects of cylindrical obstructions (common in heat exchangers) and diffuser plates were also explored. High mixing efficiency (approximately 85%) was achieved within an acceptable pressure loss (~2300 Pa) range. Gidde [26] studied a T micromixer, using three baffles (square, triangular, and circular) to achieve high mixing efficiency, and identified three flow types, namely, stratified flow (near Re = 7), eddy flow (Re = 60), and engulfing flow (Re > 100). The mixing efficiency in stratified flow is primarily due to diffusion. In the vortex state, the mixing efficiency is slightly improved due to the formation of vortices. Furthermore, as the phagocytosis process occurs, there is a sudden increase in efficiency. Rudyak and Minakov [27] optimized the T-type micromixer by changing parameters such as inlet angle, aspect ratio, speed, and slip. Arockiam et al. [28] designed and tested a micromixer with a twisty shape using basic tools found in regular labs. Mehta and Mondal [29] suggested a new micromixer design using a two-part cylinder with varying zeta potentials in opposite directions.

The construction of micromixers conventionally employs substrates composed of materials such as silicon, glass, or polydimethylsiloxane (PDMS). The fabrication methodology generally encompasses the generation of a master mold through photolithographic techniques, subsequent PDMS casting, the establishment of microchannels, and the incorporation of external components during assembly [30].

Based on the above studies of various micromixers, this paper proposes a new structure of the passive baffle micromixer to study the molecular diffusion effect and the secondary vortex effect in the microchannel by utilizing the baffle’s modulation effect on the local flow field. Through numerical simulation methods, we study the effects of four types of baffle layouts on the mixing effect and pressure drop, focus on the molecular diffusion and the secondary vortex effect in the passive baffle micromixer, and analyze the changes of the flow field in the microchannel and the performance of the micromixer under different Re, so as to provide a theoretical basis for the design of the passive micromixer.

## 2. Physical Model

**Figure 1:**
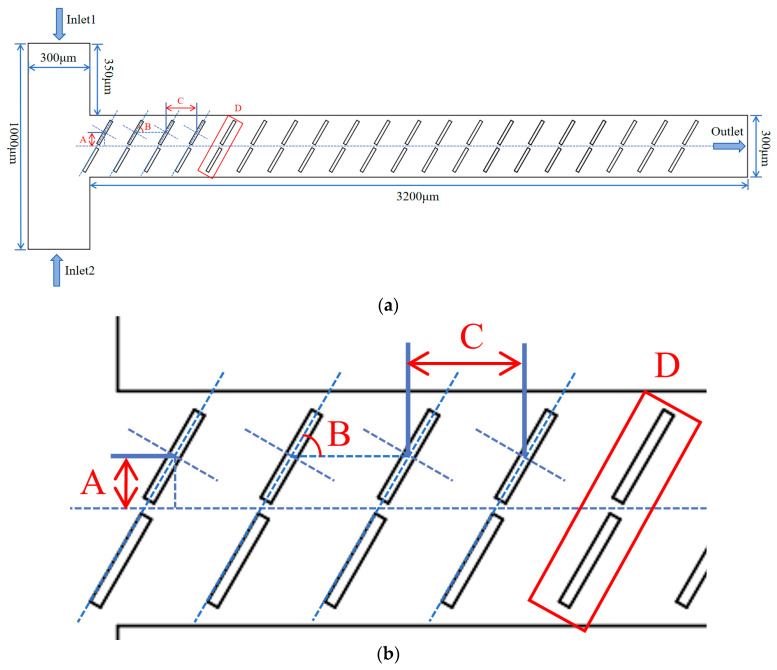
Baffle micromixer structural schematic diagram: (**a**) overall view of the baffle micromixer; And (**b**) local schematic diagram of the baffle micromixer.

A numerical model for the baffle micromixer is established as shown in Figure 1, where the inlet channel has a T-shaped configuration with two inlet widths of 300 μm each, a side length of 350 μm, and a total vertical length of 1000 μm. The outlet width of the channel is 300 μm, and the mixing length is 3200 μm.

The micromixer model incorporates 20 sets of baffles, each with a length of 130 µm and a width of 20 µm. The baffles are arranged in pairs, with one above and one below the channel. As shown in Figure 1b, A represents the distance from the center point of the rectangular baffle to the channel’s centerline, B represents the angle between the baffle and the horizontal line, C represents the distance between adjacent baffle, and D represents the number of baffle pairs. The initial model parameters are set as follows: A = 70 μm, B = 60°, C = 150 μm, and D = 20.

## 3. Research Methods and Feasibility Verification

### 3.1. The Fundamental Control Equations for Microfluidic Mixing

The numerical simulation method for studying the micromixing problem is primarily based on the continuity equation, momentum equation, and component concentration equation:

(1) ∇·V=0

(2) $$\rho V\cdot \nabla V=-\nabla p+\mu \nabla^{2}V$$

(3) $$V\cdot \nabla C=D\nabla^{2}C$$

In the equations, V represents the velocity vector, ρ is the density of the fluid, p is the pressure, μ is the absolute viscosity, C is the component concentration, and D is the component diffusion coefficient. Assumptions were made that the fluid is an incompressible Newtonian fluid, the flow state is steady, the flow structure is laminar, the channel walls exhibit no slip, and the impact of gravity on the flow is neglected. Inlet conditions were set as velocity inlet boundary conditions, and the outlet had a zero-relative-pressure pressure outlet boundary condition representing the ambient atmosphere.

The conservation equations are formulated in the following manner:

(4) $$\frac{\delta (\rho Y_{i})}{\delta x}+\nabla \cdot (\rho \upsilon Y_{i})=\nabla J_{i}+R_{i}+S_{i}$$

where $R_{i}$ is the net generation rate of chemical reactions, and $S_{i}$ represents additional generation rates caused by discrete phases and user-defined source terms. Two inlet sample concentrations were specified as C1 = 1 mol/L and C2 = 0 mol/L. The fluid density *ρ* was set to 10^3^ kg/m^3^, and the dynamic viscosity *µ* was 10^−3^ Pa·s.

Micromixing occurs within a square-wave-shaped channel, independent of the dimensions of the inlet section and Reynolds number Re. The Reynolds number Re=Ul/υ is used to characterize fluid mixing within the square-wave channel, where *U* is the average fluid velocity in the T-shaped channel, l is the hydraulic diameter of the rectangular channel (l=2(a×b)/(a+b)), and υ is the kinematic viscosity. In both experiments and numerical simulations, Re is controlled within the range of 0.1 to 100, ensuring laminar flow. The mixing intensity can be used to assess the degree of fluid mixing, and the calculation formula is as follows:

(5) $$M=\left(1-\sqrt{\frac{1}{n}\sum_{i=1}^{n}{\left(\frac{H_{i}-H_{m}}{H_{m}}\right)}^{2}}\right)\times 100\%$$

where M is the mixing intensity, n is the total number of sampling points, $H_{i}$ is the molar fraction distribution over the entire cross-section at the outlet of the micromixer, and $H_{m}$ is the average molar fraction. The calculated M represents the variation range of the mixing intensity at the outlet cross-section of the micromixer, ranging from 0% (completely unmixed) to 100% (completely mixed).

The Q criterion, proposed by Hunt and others, is used to describe the vortex characteristics by considering the relative balance between the strain rate and the magnitude of vorticity. The calculation formula is as follows:

(6) $$Q=\frac{1}{2}({\left\|\Omega \right\|}^{2}-{\left\|S\right\|}^{1})$$

(7) $$S=\frac{1}{2}(\frac{\delta_{ui}}{\delta_{xj}}+\frac{\delta_{uj}}{\delta_{xi}})$$

(8) $$\Omega =\frac{1}{2}(\frac{\delta_{ui}}{\delta_{xj}}-\frac{\delta_{uj}}{\delta_{xi}})$$

Here, S and Ω represent the symmetric and antisymmetric parts, respectively, of the velocity gradient tensor matrix Δu. When vortices are present in the flow field, the Q criterion is greater than 0. Conversely, when there is deformation at the fluid interface, the Q criterion is less than 0. The magnitude of the Q criterion also reflects the extent of the vortices and deformation in the flow field.

The efficacy of the micromixer is assessed through the utilization of the mixing cost (*MC*), which is articulated in the subsequent expression:

(9) $$MC=\frac{\Delta p/\rho u_{mean}^{2}}{M\times 100},$$

where Δ*p* is the pressure load between the inlet and the outlet, and $u_{mean}^{\;}$ represents the average velocity at the outlet.

### 3.2. Numerical Simulation Methods and Boundary Condition Settings

This paper conducted fluid dynamics analysis on the mixing units of the micromixer, investigating the influence of baffles within the passive micromixer on fluid molecular diffusion and the secondary vortex effect. Numerical calculations were performed using the COMSOL Multiphysics 6.0 fluid simulation software. The geometric model of the baffle micromixer was established, and the finite element method was employed to solve the fluid flow field and mixing effects inside the baffle micromixer. Assumptions were made that the fluid is an incompressible Newtonian fluid, the flow state is steady, the flow structure is laminar, the channel walls exhibit no slip, and the impact of gravity on the flow is neglected. Inlet conditions were set as velocity inlet boundary conditions, and the outlet had a zero-relative-pressure pressure outlet boundary condition representing the ambient atmosphere. Two inlet sample concentrations were specified as C1 = 1 mol/L and C2 = 0 mol/L. The fluid density *ρ* was set to 103 kg/m^3^, and the dynamic viscosity *µ* was 10^−3^ Pa·s.

### 3.3. Mesh Independence Verification

**Figure 2:**
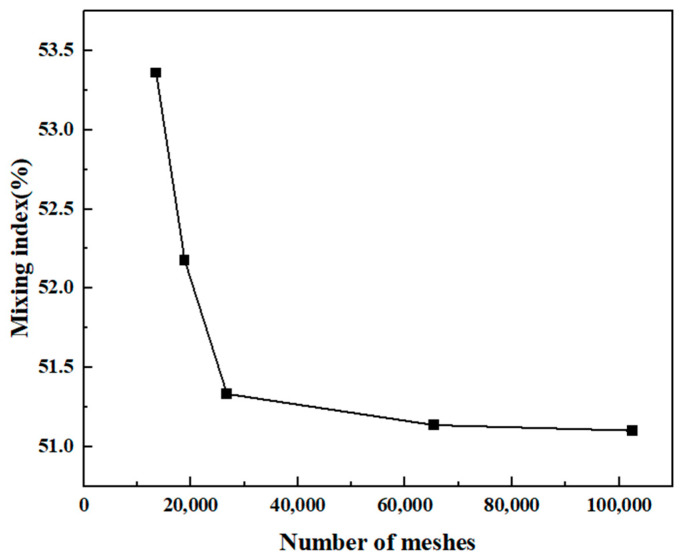
Mesh independence verification.

The simulation results vary with different numbers of meshes, and, in order to improve the simulation accuracy while saving computational time, a comparative analysis of the mixing efficiency of the micromixer was conducted under five different mesh densities (“Coarse” (13,546), “Normal” (18,870), “Fine” (26,757), “Finer” (65,473), and “Extra Fine”). As seen in Figure 2, the mixing efficiency gradually decreases with an increase in the number of meshes. For the mesh densities “Fine” and “Finer”, the mixing efficiencies are 51.3% and 51.1%, respectively, with little difference. However, the computation time is shorter for the mesh density “Fine”. Therefore, “Fine” was chosen for the numerical study.

## 4. Results and Discussion

### 4.1. The Impact of the Vertical Displacement of Baffles on the Mixing and Flow Characteristics of the Solution

In the T-shaped channel of the baffle micromixer, the vertical distance of the baffle from the horizontal centerline affects the mixing and flow characteristics of the solution. In the initial model, the vertical displacement of the baffle, denoted as A, is set to 70 μm. To achieve better mixing efficiency, additional mixing models were created with A, set to 80 μm and 90 μm, respectively. The baffles follow an odd-up, even-down layout, where baffles in odd-numbered arrays move upward, and those in even-numbered arrays move downward.

**Figure 3:**
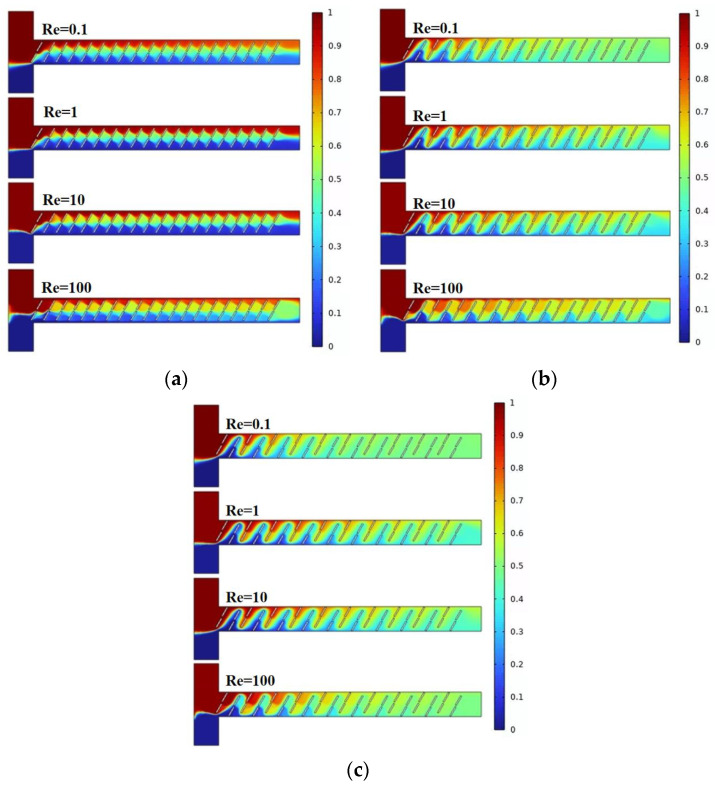
Mixing concentration distribution of baffle micromixer at different vertical displacements: (**a**) A = 70 μm; (**b**) A = 80 μm; and (**c**) A = 90 μm.

Mixing efficiency is a crucial performance metric for the micromixer. Figure 3 illustrates the mixing effects of the micromixer with three different vertical displacements of the baffles. Different colors represent different solute concentrations, with the best mixing achieved when the solute concentration at the microchannel outlet is 0.5 mol/L. As observed in the figure, with an increase in the vertical displacement of the baffles, the overall mixing efficiency improves. For a given displacement, mixing effects at Re = 0.1 and Re = 100 are generally better than those at Re = 1 and Re = 10.

**Figure 4:**
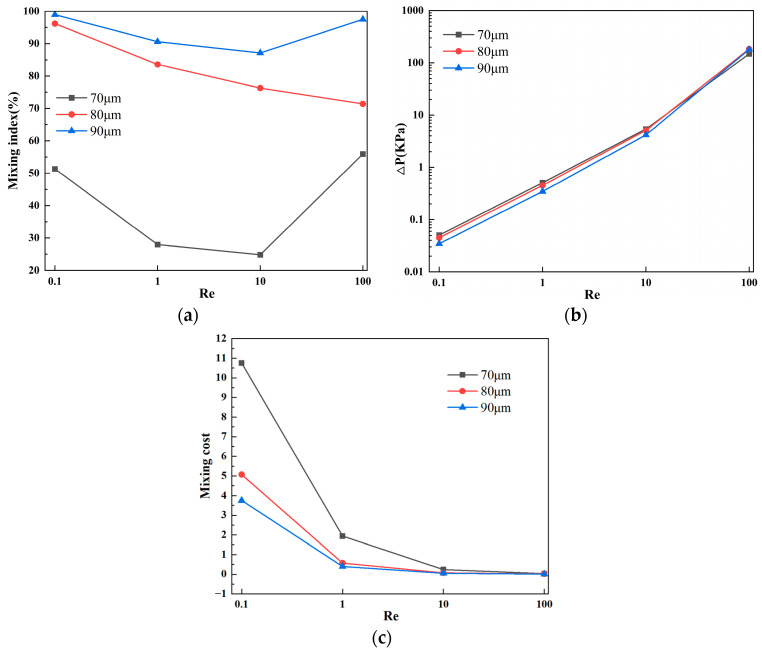
Mixing efficiency and pressure drop variation at different vertical displacements in the micromixer: (**a**) mixing efficiency curve; (**b**) pressure drop curve; and (**c**) mixing cost.

Figure 4a presents the mixing efficiency curves at the outlet of the micromixer with different vertical displacements of the baffles under four flow velocities. Two samples with different concentrations are introduced into the two inlets of the micromixer. The horizontal axis represents Re, and the vertical axis shows the mixing efficiency. From Figure 4a, it can be visually observed that, among the three structures, the micromixer achieves the best overall mixing efficiency when the vertical displacement of the baffles is 90 μm. Considering various factors affecting the performance of the micromixer, the influence of pressure drop cannot be ignored. Figure 4b illustrates the pressure drops for the three structures, and it is evident that the micromixer with a vertical displacement of 90 μm has a significantly lower pressure drop than the other two structures. However, at Re = 100, this trend slightly changes, and the pressure drops for the three micromixer structures are 147.8 kPa, 184.5 kPa, and 180.1 kPa, respectively. When the Re number is low, the pressure drop variation is not pronounced, which may be related to the low flow velocity at the inlet. Figure 4c demonstrates the mixing cost of the micromixers. It can be seen that the mixing cost diminishes with an increase in Re. Similarly, when the vertical displacement of the baffles is 90 μm, the mixing cost of the micromixer is minimized.

**Figure 5:**
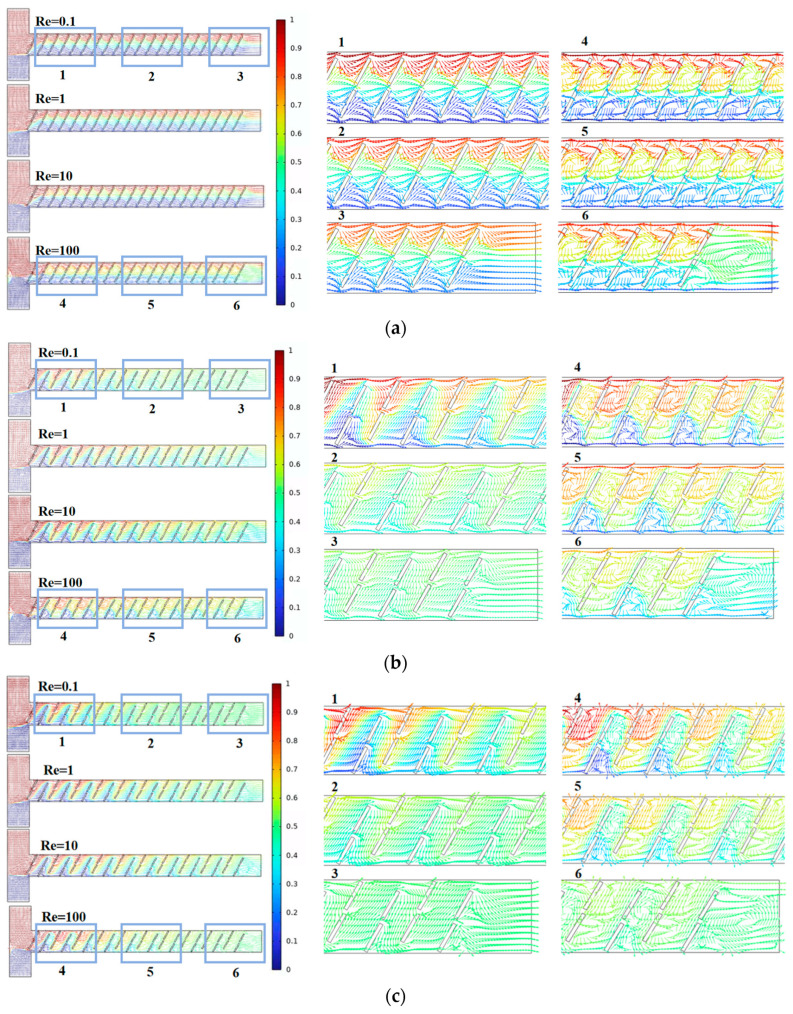
Velocity vector distribution within the baffle micromixer at different vertical displacements: (**a**) A = 70 μm; (**b**) A = 80 μm; and (**c**) A = 90 μm.

Next, we analyzed the flow field states inside the micromixer channels in conjunction with different Re and fluid diffusion modes. Figure 5 represents streamline plots for the three micromixer channels, where different colors indicate different fluid concentrations, with a value of 0.5 representing complete mixing. From the above analysis, the micromixer demonstrates good mixing efficiency at both low and high Re, warranting further investigation. By comparing the streamline plots for six cross-sections of each micromixer, it is observed that, when Re = 0.1, the dominant mode of fluid diffusion in the micromixer is molecular diffusion. At this point, the fluid velocity is relatively low, and the fluid exhibits a steady laminar flow. The mixing efficiency is related to the total path of fluid flow in the microchannel, where longer flow paths result in longer residence times, promoting mixing. When Re = 100, the area and velocity of the double-vortex micro-vortices distributed along the main channel sides are larger. The chaotic convection within the micromixer becomes stronger, leading to a more uniform distribution of fluid concentrations. In summary, the micromixer with A = 90 μm can achieve a larger mixing index while ensuring a smaller pressure drop, making it more advantageous in terms of performance.

### 4.2. Effect of Baffle Rotation Angle on Solution Mixing and Flow Characteristics

Based on the discussion in Section 4.1, we selected the micromixer structure with A = 90 μm and further investigated the mixing conditions for baffle angles B = 45°, B = 60°, and B = 75°. The baffles follow an odd-down, even-up layout, where odd-numbered arrays change the angle of the baffles below the centerline, and even-numbered arrays change the angle of the baffles above the centerline.

**Figure 6:**
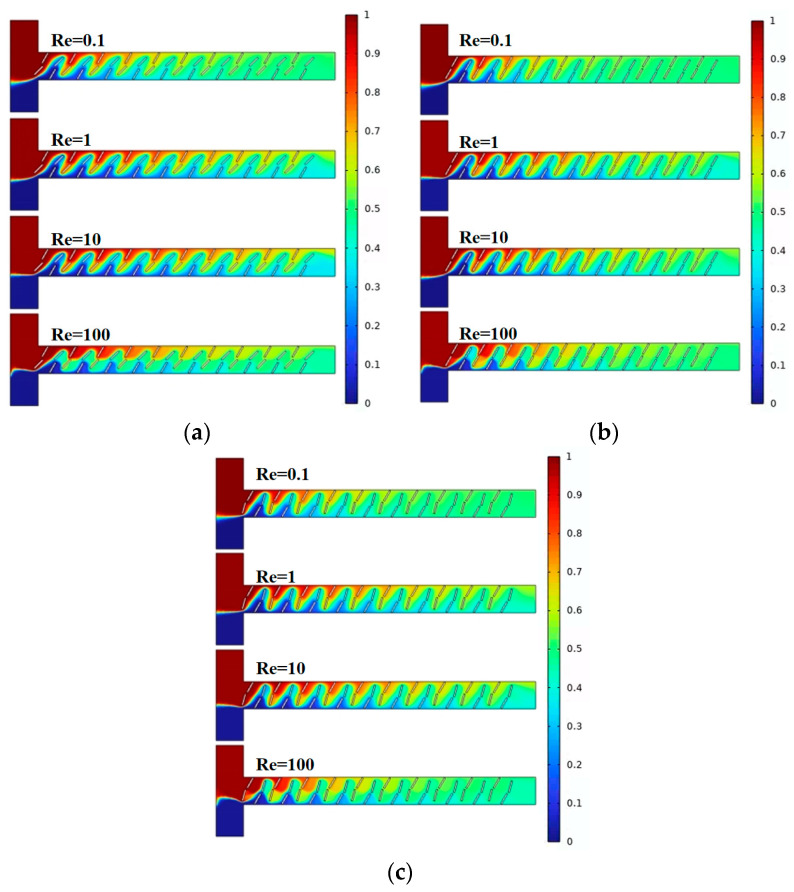
Mixing concentration distribution of baffle micromixer at different rotation angle: (**a**) B = 45°; (**b**) B = 60°; and (**c**) B = 75°.

**Figure 7:**
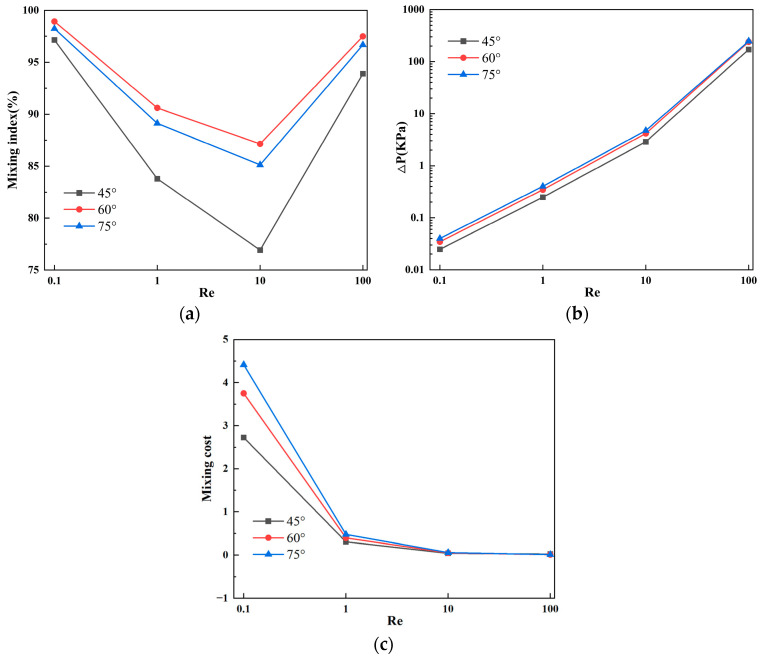
Mixing efficiency and pressure drop changes of micromixer at different rotation angles: (**a**) mixing efficiency curve; (**b**) pressure drop curve; and (**c**) mixing cost.

Figure 6 illustrates the mixing effects of the micromixer with three different baffle arrangement angles. Different colors represent different solute concentrations, with the best mixing achieved when the solute concentration at the microchannel outlet is 0.5 mol/L. Combined with Figure 7a, it can be observed that, as the Reynolds number increases, the mixing efficiency of baffles with different angles generally shows a trend of decreasing first and then increasing. When the baffle angle is 60°, the mixing efficiency is the highest. Figure 7b displays the pressure drops for the three structures, indicating that a larger baffle rotation angle corresponds to a higher pressure drop. At Re = 100, the pressure drops for the three micromixer structures are 171.4 kPa, 240.2 kPa, and 247.9 kPa, respectively. At Re = 0.1, the pressure drop changes are not significant, measuring 0.0248 kPa, 0.03457 kPa, and 0.04006 kPa, respectively. Figure 7c represents the mixing cost, from which it can be concluded that the mixing cost diminishes with an increase in Re and that the 45° baffle angle micromixer exhibits the lowest mixing cost. At Re = 0.1, the mixing costs for the three micromixer structures are 2.72726, 3.7523, and 4.41811, respectively. At Re = 100, the mixing costs are 0.02519, 0.0142, and 0.01321.

**Figure 8:**
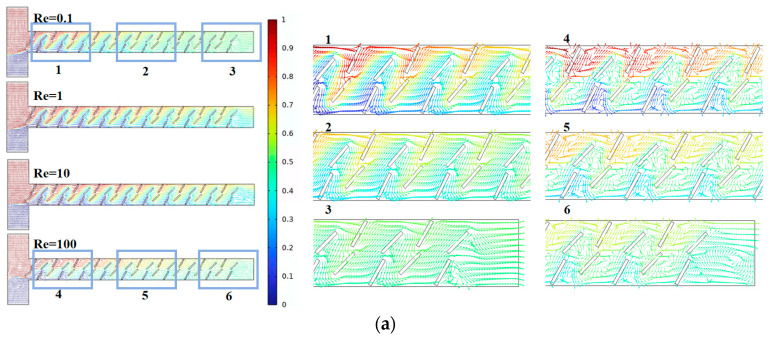

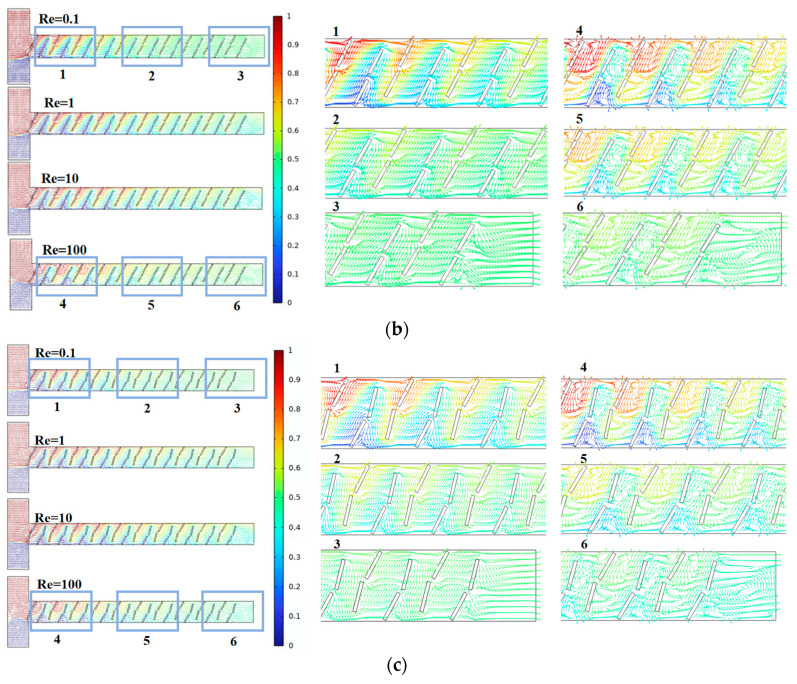
Velocity vector distribution within the baffle micromixer at different rotation angles: (**a**) B = 45°; (**b**) B = 60°; and (**c**) B = 75°.

Figure 8 represents streamline plots inside the channels of the three micromixers, where different colors indicate different fluid concentrations, with a value of 0.5 indicating complete mixing. By comparing the streamline plots for six cross-sections of each micromixer, it can be observed that, when the baffle rotation angle is 60°, the fluid is more stable at Re = 0.1, and the mixing effect is better at cross-sections 2 and 3 compared to the other two structures. At Re = 100, the vortex region is more pronounced, significantly enhancing chaotic convection in the micromixer.

Overall, the micromixer with B = 60° exhibits the maximum mixing efficiency and a moderate pressure drop, making it a more versatile choice in terms of performance.

### 4.3. Effect of Baffle Horizontal Spacing on Solution Mixing and Flow Characteristics

Based on the discussion in Section 4.2, we selected the micromixer structure with B = 60° and further investigated the mixing conditions for baffle horizontal spacings C = 100 μm, C = 130 μm, and C = 150 μm.

**Figure 9:**
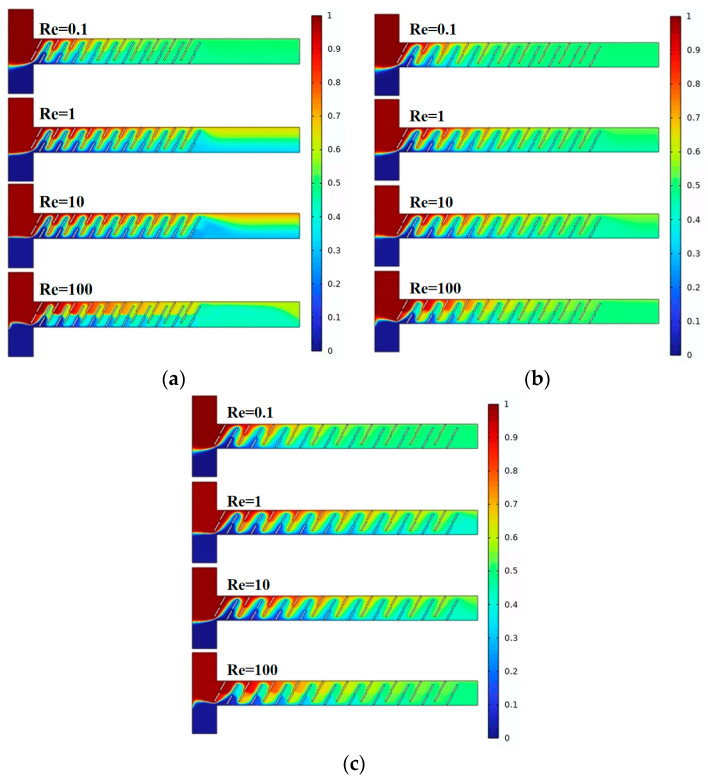
Mixing concentration distribution of baffle micromixer at different horizontal spacing: (**a**) C = 100 μm; (**b**) C = 130 μm; and (**c**) C = 150 μm.

**Figure 10:**
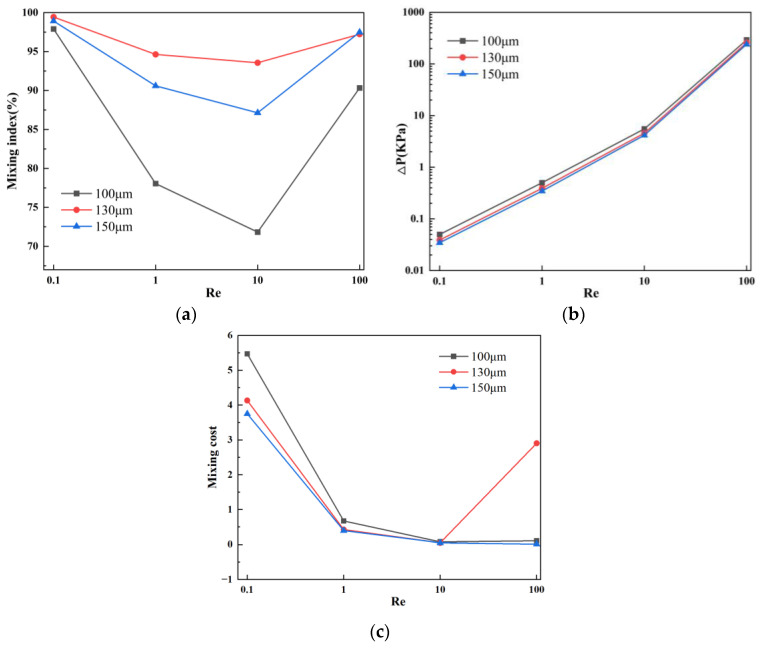
Mixing efficiency and pressure drop changes of micromixer at different horizontal spacing: (**a**) mixing efficiency curve; (**b**) pressure drop curve; and (**c**) mixing cost.

Figure 9 and Figure 10a show the mixing effect of the micromixer baffles at three horizontal spacings. It can be seen from the figure that the mixing effect of C = 130 μm is greater than that of C = 100 μm. As the horizontal displacement of the baffle increases, the mixing effect when C = 150 μm is smaller than 130 μm. The mixing effect when Re = 0.1 and Re = 100 is better than Re = 1 and Re = 10. Figure 10b shows the pressure drops of the three structures. The larger the horizontal distance between the baffles, the smaller the pressure drop. When Re = 100, the pressure drops of the three structures of micromixers are 291.4 kPa, 258.1 kPa, and 240.2 kPa, respectively. When the Re number is low, the pressure drop changes are not obvious, which are 0.05004 kPa, 0.03894 kPa, and 0.03457 kPa, respectively. Figure 10c reveals that, across different Re, the mixing cost is minimized when C = 150 μm. Additionally, at Re = 100, the mixing cost increases for C = 130 μm, with respective values for the three structures being 0.10683, 2.90532, and 0.0142.

**Figure 11:**
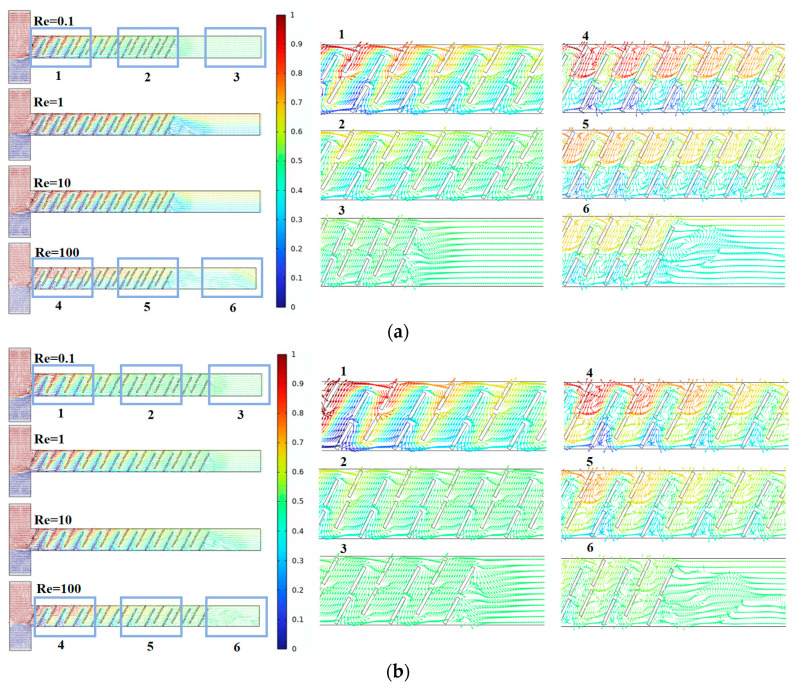

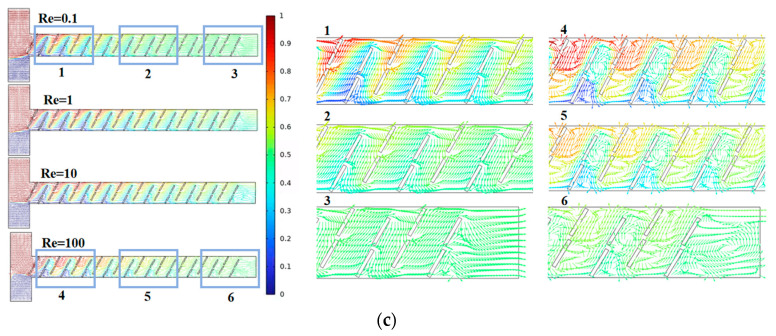
Velocity vector distribution within the baffle micromixer at different horizontal spacing: (**a**) C = 100 μm; (**b**) C = 130 μm; and (**c**) C = 150 μm.

Figure 11 represents streamline plots inside the channels of the three micromixers, where different colors indicate different fluid concentrations, with a value of 0.5 indicating complete mixing. When the baffle spacing increases from 100 μm to 130 μm, the individual active area of the fluid expands, enhancing mixing efficiency. However, when the baffle spacing further increases to 150 μm, the excessively large active area weakens the effect of changing fluid trajectories, resulting in decreased mixing efficiency. Overall, the micromixer with C = 130 μm demonstrates advantages in performance, providing a larger mixing index while ensuring a smaller pressure drop.

### 4.4. Effect of Baffle Logarithm on Solution Mixing and Flow Characteristics

Combining the discussion in Section 4.3, we chose the micromixer structure with C = 130 μm and further investigated the mixing conditions for baffle horizontal spacings D = 10 groups, D = 15 groups, and D = 20 groups.

**Figure 12:**
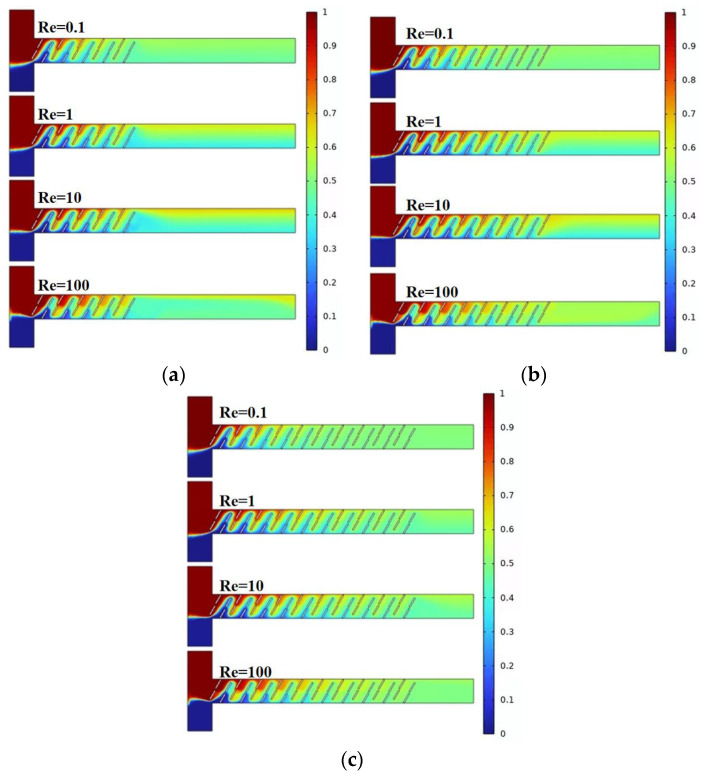
Mixing effect of micromixer with different number of baffles: (**a**) D = 10 pairs; (**b**) D = 15 pairs; and (**c**) D = 20 pairs.

**Figure 13:**
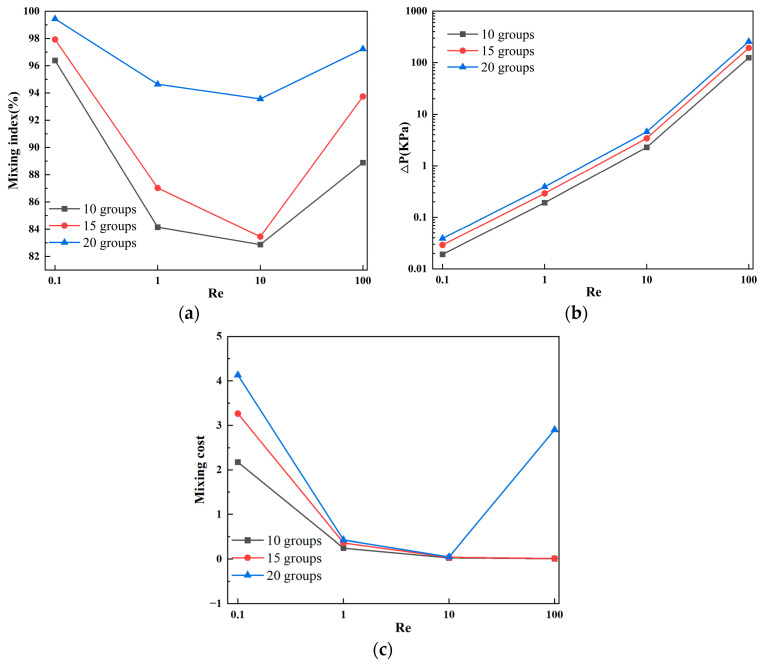
Mixing efficiency and pressure drop changes of micromixer with different number of baffles: (**a**) mixing efficiency curve; (**b**) pressure drop curve; and (**c**) mixing cost.

Figure 12 and Figure 13a provide the simulation results for the three different numbers of baffles. With an increase in the number of baffle groups, the fluid undergoes continuous compression and separation in the microchannel, significantly improving the mixing efficiency. Figure 13b shows the pressure drops for the three structures, indicating that a greater number of baffle groups corresponds to a larger pressure drop. Especially under high Reynolds number conditions, the generation of vortices and other complex flow phenomena significantly increases the pressure drop. In fact, a high-pressure drop is an unavoidable drawback of micromixers. Figure 13c indicates that an increase in the number of baffle groups enhances mixing efficiency; however, the elevated pressure drop results in a higher mixing cost. When the number of baffle groups is 20, the mixing cost is maximized, and, under the high Reynolds number condition of Re = 100, the mixing cost increases. At this point, the mixing costs for the three structures are 0.00945, 0.01556, and 2.90532, respectively.

**Figure 14:**
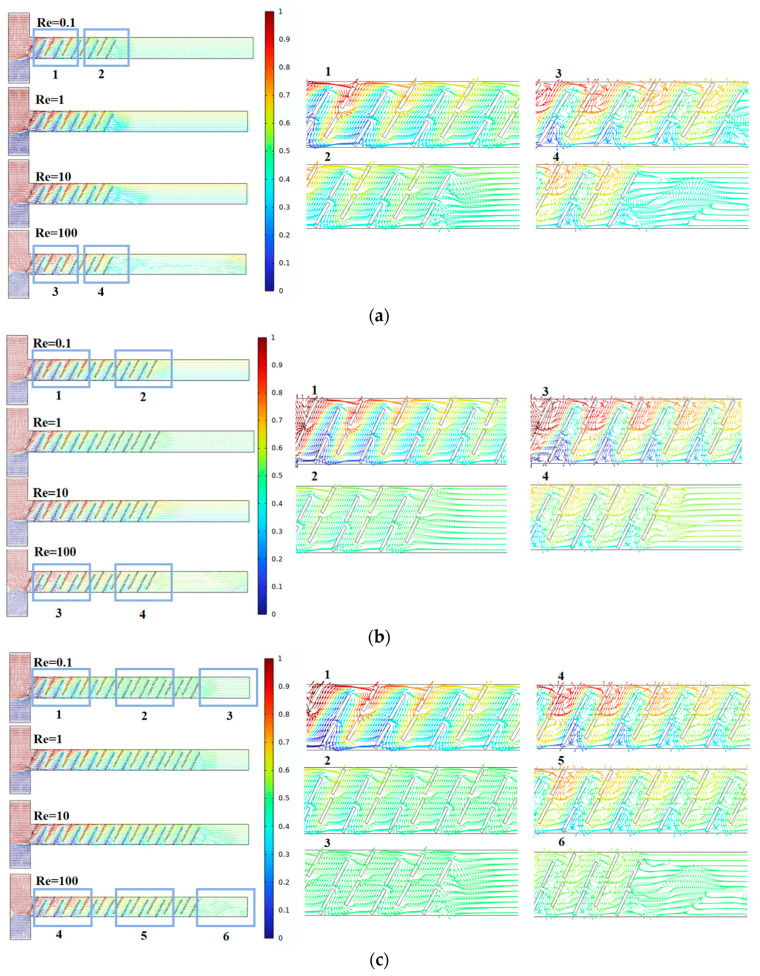
Velocity vector distribution within the baffle micromixer at different quantities: (**a**) D = 10 groups; (**b**) D = 15 groups; and (**c**) D = 20 groups.

Figure 14 represents the velocity vector distribution inside the channels of the three micromixers, where different colors indicate different fluid concentrations, with a value of 0.5 indicating complete mixing. As the number of baffle groups increases, the mixing time extends at low Re, enhancing the mixing effect. At high Re, in addition to high-intensity vortices, there are also many less apparent small vortices. This is of significant importance for micromixers aiming to improve mixing efficiency. Overall, the micromixer with D = 20 groups demonstrates the optimal mixing efficiency and a higher cost-performance ratio.

## 5. Conclusions

This paper addresses the challenges of low mixing efficiency and integration in passive micromixers. Utilizing baffles to control the local flow field, the study conducts a numerical simulation analysis to investigate the impact of different baffle structures on the mixing index and pressure drop. The main conclusions are as follows:

(1) The optimal mixing effect is achieved when the baffle vertical displacement is 90 μm, the baffle angle is 60°, horizontal spacing is 130 μm, and the number of baffles is 20 groups. At Re = 0.1, the mixing efficiency reaches 99.4%, and, at Re = 100, the mixing efficiency is 97.2%.
(2) With the gradual increase of Re, the mixing efficiency shows a trend of decreasing first, and then increasing. When Re = 0.1, the dominant mode of fluid diffusion in the micromixer is molecular diffusion, and the mixing efficiency is related to the total path of fluid flow in the microchannel. As Re increases to 100, the area and velocity of the double vortex micro-eddies distributed along the main flow channel increase, enhancing chaotic convection in the micromixer and resulting in a more uniform concentration distribution of fluids.
(3) The increase in mixing efficiency does not necessarily lead to a proportional increase in pressure drop in the micromixer. This finding suggests a new approach to structural optimization, enabling a high mixing efficiency while maintaining a relatively small pressure drop. This insight provides a reference for the design and development of micromixers with efficient mixing functionality for the preprocessing part in microfluidic analysis and micro-total analysis.

The advantages of the paper are the achievement of high mixing efficiency, and the relationship between Re and mixing efficiency, as well as the pattern between the pressure drop mixing efficiency. In the future, we will further explore micromixers with a high mixing efficiency with little pressure drop. The attainment of heightened mixing efficiency in micromixer design, concomitant with the mitigation of pressure drop, necessitates the judicious optimization of microchannel geometry, the adept control of fluid flow rates, and the discerning incorporation of passive or active mixing methodologies. The utilization of advanced fabrication techniques such as 3D printing to engender intricate channel architectures, the contemplation of recirculation zones, and recourse to computational simulations for systematic design exploration stand as pivotal measures in achieving an optimal equilibrium between mixing efficacy and minimal pressure drop.

## Data Availability

Data are contained within the article.
